# Unlocking Drought-Induced Tree Mortality: Physiological Mechanisms to Modeling

**DOI:** 10.3389/fpls.2022.835921

**Published:** 2022-04-04

**Authors:** Ximeng Li, Benye Xi, Xiuchen Wu, Brendan Choat, Jinchao Feng, Mingkai Jiang, David Tissue

**Affiliations:** ^1^College of Life and Environmental Science, Minzu University of China, Beijing, China; ^2^Hawkesbury Institute for the Environment, Western Sydney University, Richmond, NSW, Australia; ^3^Ministry of Education Key Laboratory of Silviculture and Conservation, Beijing Forestry University, Beijing, China; ^4^State Key Laboratory of Earth Surface Processes and Resource Ecology, Beijing Normal University, Beijing, China; ^5^College of Life Sciences, Zhejiang University, Hangzhou, China; ^6^Global Centre for Land-based Innovation, Western Sydney University, Richmond, NSW, Australia

**Keywords:** drought, tree mortality, hydraulic failure, carbohydrates, functional traits, plant hydraulics, land surface models

## Abstract

Drought-related tree mortality has become a major concern worldwide due to its pronounced negative impacts on the functioning and sustainability of forest ecosystems. However, our ability to identify the species that are most vulnerable to drought, and to pinpoint the spatial and temporal patterns of mortality events, is still limited. Model is useful tools to capture the dynamics of vegetation at spatiotemporal scales, yet contemporary land surface models (LSMs) are often incapable of predicting the response of vegetation to environmental perturbations with sufficient accuracy, especially under stressful conditions such as drought. Significant progress has been made regarding the physiological mechanisms underpinning plant drought response in the past decade, and plant hydraulic dysfunction has emerged as a key determinant for tree death due to water shortage. The identification of pivotal physiological events and relevant plant traits may facilitate forecasting tree mortality through a mechanistic approach, with improved precision. In this review, we (1) summarize current understanding of physiological mechanisms leading to tree death, (2) describe the functionality of key hydraulic traits that are involved in the process of hydraulic dysfunction, and (3) outline their roles in improving the representation of hydraulic function in LSMs. We urge potential future research on detailed hydraulic processes under drought, pinpointing corresponding functional traits, as well as understanding traits variation across and within species, for a better representation of drought-induced tree mortality in models.

## Introduction

The survival of forests around the globe is increasingly threatened by climatic extremes (Brodribb et al., 2020; Bennett et al., 2021; Gora and Esquivel-Muelbert, 2021). Massive forest dieback induced by climate change type drought has been recorded on every vegetated land within the past few decades (Adams et al., 2009; Allen et al., 2010, 2015). These events will become more common given that climate projections indicate that future drought episodes will be typified by increased frequency, severity, and duration (Adams et al., 2009; Carnicer et al., 2011; Dai, 2013). Forests cover approximately 30% of the land surface area and provide numerous crucial ecological, economic and social benefits. Globally, forests store *ca*. 45% of the carbon in terrestrial ecosystems and sequester approximately 25% of annual anthropogenic carbon emissions each year (Bonan, 2008; Pan et al., 2011). In addition, forests can regulate the terrestrial energy budget and hydrological cycle by modifying surface albedo and evapotranspiration, which exert strong control over global carbon cycle. Forest mortality can therefore significantly influence ecosystem structure and function, resulting in cascading negative effects on biochemical and biophysical cycles, consequently generating a positive feedback to the climate (Adams et al., 2010; Anderegg et al., 2013).

Elucidating the spatial-temporal pattern of imminent mortality events is essential for ameliorating the detrimental consequences of tree death triggered by drought. Land surface models (LSMs) with mechanistic representations of vegetation and soil processes are an efficacious tool for predicting future plant dynamics (Geary et al., 2020). These models are capable of simulating growth, mortality, and reproduction of vegetation and are often coupled with climate models to predict the dynamics of biosphere-atmosphere interactions at large scales (Fisher et al., 2014). Early LSMs often poorly performed when simulating tree mortality events caused by drought, as evidenced by the existence of offsets between model outcomes and field observations (McDowell et al., 2013; Bugmann et al., 2019; Thrippleton et al., 2021). While subsequent progress in physiology research has led to updated model structure and components, thereby enabling better predictions of ecosystem fluxes as well as vegetation dynamics under drought with improved accuracy (De Kauwe et al., 2020; Sabot et al., 2020; Wang et al., 2021), predicting drought-related tree mortality remains a significant shortcoming of current LSMs (Trugman, 2021).

More accurate simulation of drought-induced tree mortality events requires detailed knowledge of the mechanisms through which drought affects plant physiology and the cascading effects on plant carbon and water status. Empirical models exist, but they often lack theoretical underpinnings; thus their predictive power for novel conditions or subjects is limited. Process-based models can overcome these weaknesses by representing biological processes in detail. Current experimental evidence points to the crucial role of plant hydraulic traits in explaining the pattern of drought-induced tree mortality (Anderegg et al., 2016; Zhu et al., 2018; Chen et al., 2019, 2021a; Powers et al., 2020; Nolan et al., 2021), which subsequently motivated the development and integration of plant hydraulic modules into LSMs (De Kauwe et al., 2020; Eller et al., 2020; Sabot et al., 2020; López et al., 2021). Nonetheless, physiological processes driving hydraulic dysfunction are incompletely integrated into these models, mainly because of knowledge gaps surrounding the physiological mechanisms of drought response, as well as inadequate empirical datasets enabling adequate model parameterization (Meir et al., 2015; Hartmann et al., 2018). Hence, modelers must employ largely unvalidated assumptions such as leaf phenology when drought strikes, lethal threshold during drought or post-drought recovery of hydraulic function, which consequently produce unrealistic outcomes (McDowell et al., 2013; Xu et al., 2016; De Kauwe et al., 2020), e.g., model suggests that trees in South-East Australia forests did not approach lethal water potential (De Kauwe et al., 2020), despite large-scale forest mortality was substantiated by field observation (Nolan et al., 2021).

In light of the modeling need, this review attempts to provide a brief overview of the current understanding of mechanisms leading to tree mortality under drought stress, with particular emphasis on carbon and hydraulic functions. Next, the development of hydraulic failure under drought stress, together with current understanding about key traits modulating this process, is outlined and discussed. We suggest that incorporating these hydraulic traits would improve the predictability in terms of mortality events during drought of process-based models. Finally, coordination among traits is discussed in the context of facilitating model parameterization. Here, we emphasize that this review does not attempt to provide a comprehensive summary of the gaps between data and models; such a summary must be interdisciplinary and requires iterative collaborations between modelers and experimentalists. Rather, our aim is to facilitate such an advancement by providing a physiological interpretation of the merit of functional traits, especially hydraulic traits involved in drought tolerance, to improve the predictive power of vegetation models under climate change. Finally, the matter of scale is a crucial but challenging consideration when incorporating plant hydraulic traits to predict drought-induced mortality in LSMs. While tree death at the individual level can be quantitatively determined *via* better representation of plant physiology, mortality events at the larger scales (e.g., population and landscape) are much more complex, involving interactions among individuals within and across species, heterogeneous plant-environment feedback across the landscape, and stochasticity to account for other co-variates that are difficult to capture in a typical LSM (e.g., insect and disease effects). Given that our overall objective is to frame a pathway linking plant hydraulic trait with improved predictive power in the models, the current review will focus exclusively on attributes at organ or canopy scale, while characteristics at other spatial scales are beyond the scope of discussion.

## The History of Tree Mortality Research: Physiological Mechanisms

Four decades ago, Manion (1981) proposed a slow decline hypothesis to explain the progress of tree mortality. In this theory, tree death begins with a moderate long-term stress that predisposes trees to mortality risk, which is intensified by a short-term severe stress, and death eventually occurs due to a contributing factor. Early modeling often builds on this original framework, with particular emphasis being placed on the role of carbon balance in tree mortality (McDowell et al., 2011; Sevanto and Xu, 2016). Based on the many physiological processes documented in trees approaching death under drought stress, McDowell et al. (2008) put forward two physiological hypotheses that explain and generalize mechanisms underlying this phenomenon. The *carbon starvation* (*CS*) hypothesis suggests that downregulated stomatal conductance during chronic, moderate drought stress will lead to reduced carbon assimilation. As plants still require photosynthate to fuel metabolic processes such as respiration and osmoregulation, limited carbon supply, and the continued demand for it will eventually deplete plant carbon storage (e.g., non-structural carbohydrates, NSCs), which in turn causing tree death due to carbon starvation. Alternatively, the *hydraulic failure* (*HF*) hypothesis suggests that water transport failure-induced desiccation is the primary cause of tree mortality under drought stress. According to the air-seeding hypothesis (Zimmermann, 1983), when xylem water transport is operating under greater tension during acute drought due to high evaporative demand and/or decreased soil water availability, air will be aspirated from air-filled vessels into adjacent water-filled, functional vessels through inter-conduit pits, forming air bubbles, and consequently creating emboli that break the continuity of water transport in xylem. Embolized conduits are unable to transport water to distal organs and are unlikely to recover over short periods (Brodribb et al., 2010; Choat et al., 2015; Li et al., 2018b; Choat et al., 2019), eventually leading to desiccation and death at multiple organizational levels (i.e., cell, tissue, organ, and whole plant). It is worth noting that hydraulic failure does not exclusively occur in the vascular system of plants, but can also arise at the plant-soil interface as the hydraulic pathway connecting roots and soil is interrupted by air (Sperry et al., 1998). However, mortality under drought is still finalized by hydraulic failure of vascular system in this scenario.

Given the intimate link between carbon and water metabolism, the binary theory of tree mortality mechanisms during drought stress is apparently oversimplified. In recognition of the interdependency between *CS* and *HF*, an integrated mechanism was further proposed (McDowell et al., 2011). In this updated theory, *CS* and *HF* are synergistic and interrelated through a “carbon-hydraulic feedback loop.” During drought stress, rates of carbon assimilation progressively decrease due to stomatal closure, consequently reducing the tissue non-structural carbohydrates content, which act as osmolytes facilitating water absorption by maintaining the water potential gradient between plant and soil. Inadequate water supply will result in increased xylem tension and degree of embolism, which diminishes hydraulic conductivity and leads to intensified physiological water deficit. This will ultimately feedback to leaves, causing enhanced stomatal closure or leaf shedding, which in turn exacerbates the NSCs deficiency. This integrated framework is generally supported, as both shifted tissue NSCs and xylem conductivity are often observed concomitantly in plants killed by drought (Adams et al., 2017). Therefore, pure CS or HF may not exist. Rather, *CS* and *HF* are likely the two endpoints of the “mortality mechanism spectrum”. However, it is still necessary to identify the primary contributing factors for drought-induced tree mortality given that models require quantitative information on the tipping point where recovery is impossible, so as to accurately predict the dynamics of tree mortality under different environmental conditions. Incorporating both mechanisms will likely add extra complexity to computation without much improvement of predictive power.

## Non-structural Carbohydrates Facilitate the Integrity of Xylem Water Transport

Carbohydrate may be involved in the process of drought-related tree mortality through its effects on plant hydraulics, particularly by lowering the risk of xylem embolism. In support of this theoretical expectation, a strong relationship between the percentage loss of xylem conductivity (PLC) and the extent of NSC depletion has been observed (Trifilò et al., 2017; Tomasella et al., 2019). It has long been acknowledged that NSCs contribute to osmotic regulation, which may lower the risk of hydraulic failure by increasing plant water uptake (De Roo et al., 2020). For example, by manipulating tissue NSCs, O’Brien et al. (2014) showed that trees fed by exogenous NSCs exhibited higher survival rate during drought stress. Trees with enriched NSCs typically exhibited less negative water potential, which in turn reduced PLC, assuming xylem vulnerability to embolism was not shifted by the treatment. Alternatively, NSCs may lower the risk of xylem embolism by restraining the movement of air bubbles within the vascular system under negative pressure (De Baerdemaeker et al., 2017). It has been suggested that several carbon-based organic compounds, such as choline, amphiphilic lipids, or proteins, may act as surfactants that increase the stability of nanobubbles against increased tensile force, thereby lowering xylem vulnerability to embolism (Schenk et al., 2017). In addition, NSCs have been suggested to facilitate the repair of embolism by generating osmotic force that helps draw water from the reservoir to air-filled conduits (Liu et al., 2019) or change the embolism resistance by mediating the structure of vessels during the ontogenesis of xylem. Note that both NSC-mediated xylem refilling, and structural change requires sufficient time to occur and thus may not alter the fate for trees exposed to short-term pules of lethal drought.

## Hydraulic Dysfunction Determines Tree Death During Drought

The interplay between *CS* and *HF* is intricate, hence validating these mechanisms can be complex. In many studies, partial or complete loss of hydraulic conductivity in dead trees has been observed with little or no change in tissue carbohydrate concentration. A multi-species synthesis shows trees that died from drought typically exhibit more than 60% embolism with various degrees of carbon depletion, indicating hydraulic failure is a universal phenomenon for trees exposed to lethal drought stress (Adams et al., 2017). The role of plant hydraulics in tree death under drought is further supported by studies showing correlative relationships between hydraulic traits related to embolism resistance and mortality patterns at both regional and global scales (Anderegg et al., 2016; Li et al., 2018a; Nolan et al., 2021; Chen et al., 2021a). In comparison, experimental evidence supporting carbon starvation as the main mechanism of mortality is relatively scarce. In Sevanto et al. (2014), tissue carbohydrate content decreased by nearly 70% in dead *Pinus edulis* treated with light exclusion, while both water potential and xylem conductivity were comparable to non-stressed controls. These results indicate that protracted carbon deficiency can generate tree death independent of hydraulic impairment. However, in the same study, trees killed by drought stress showed identical loss of xylem conductivity but different degrees of carbon depletion depending on the duration of drought exposure; hence, hydraulic failure remained the decisive factor generating tree mortality during drought stress.

## Deciphering the Process of Drought-Induced Hydraulic Failure

**Figure 1:**
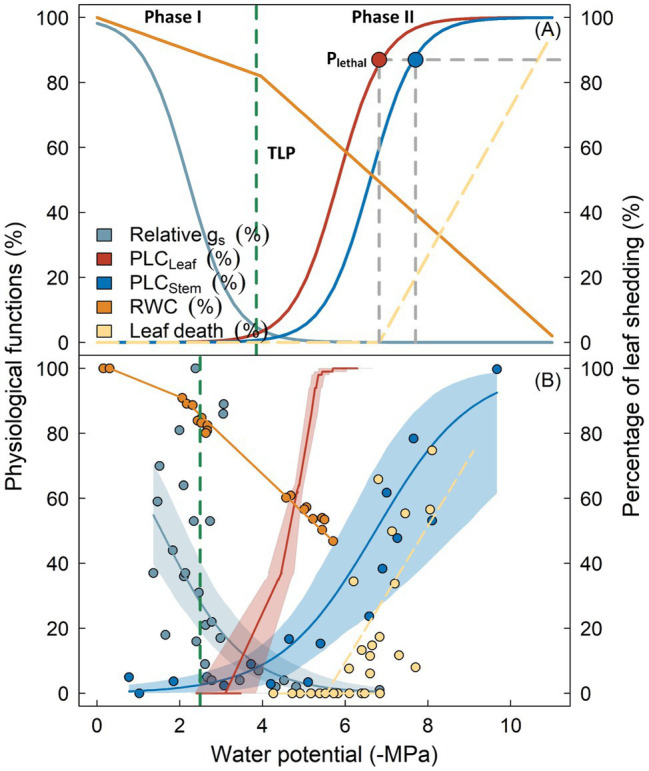
Key physiological processes following reductions in plant water potential as outlined by the biphasic framework of drought-related tree mortality (panel **A**). Physiological functions including stomatal conductance (*g*_s_, cyan), percentage loss of hydraulic conductivity in leaves (PLC_Leaf_, red) and stems (PLC_Stem_, blue), as well as branch relative water content (RWC, orange) are shown as percentage of maximum. Vertical dashed line indicates the leaf turgor loss point (TLP). Lethal water potential thresholds (P_Lethal_) for leaves and stems are indicated by red and blue circles. Transition from Phase I to Phase II occurs when stomata are fully closed, which theoretically coincides with the turgor loss (broken dashed line). Panel **(B)** shows the observed variation of these physiological processes from *Eucalyptus sideroxylon* during a dry-down experiment conducted in a common garden (Blackman et al., 2019), with shaded regions surrounding the lines denoting the 95% confidence interval of fitted curves. Similarity in the two panels indicate that the biphasic framework is generally supported by the experimental evidence. Note that TLP in panel **(B)** occurred prior to complete stomatal closure, and leaf shedding was initiated when leaf xylem was completely embolized, indicating these traits may not be robust for predicting the timing of these physiological adjustments (see text for detail).

Incorporating hydraulic failure into models requires its process to be elaborated. According to Choat et al. (2018), the development and progression of hydraulic failure under drought can be divided into two major phases (Figure 1A). During the initial phase (Phase I), drought stress develops as soil water availability declines due to decreased precipitation or depth to groundwater and can be exacerbated by an increase in the soil-atmosphere moisture gradient (i.e., vapor pressure deficit). Xylem tension becomes greater, as indicated by the continuously decreasing (i.e., more negative) maximum water potential (Ψ) of plants (e.g., predawn Ψ). Plants minimize water loss by reducing stomatal conductance until complete stomatal closure, either driven by the loss of turgor in guard cells and/or accumulation of cellular hormones (Buckley, 2019). It has been shown in many studies that the occurrence of complete stomatal closure precedes the inception of xylem embolism (Hochberg et al., 2017; Martin-StPaul et al., 2017; Creek et al., 2020). Therefore, the xylem water transport system remains largely intact during this stage, with no significant change in the hydraulic conductivity.

As drought stress persists, plant water potential slowly decreases. Roots can no longer extract water from soil due to xylem cavitation or disconnection between roots and soil, leading to decoupled water potentials between soil and plants. Once stomata are fully closed, water leaves the plant mainly through the leaf cuticle, leaky stomata, or other tissues, although with markedly reduced rates (Phase II; Duursma et al., 2019). Water will be extracted from intercellular spaces, apoplast, or living tissues (i.e., hydraulic capacitance), to meet the evaporation demand and maintain cellular water status. In some species, water loss during this stage can be further prevented by leaf shedding (Pivovaroff et al., 2014; Levionnois et al., 2020). If drought remains unalleviated, xylem water potential will ultimately exceed the threshold of embolism. Air bubbles rapidly propagate and spread within the vascular system until all vessels are embolized, with the spatial pattern of propagation depending on the vessel arrangement and connectivity (Johnson et al., 2020; Wason et al., 2021). Complete loss of water transport capacity results in desiccation of living tissue and cellular death, including meristematic cells that govern post-drought resilience, finally resulting in whole tree mortality (Li et al., 2016; Mantova et al., 2021).

## Parameterizing Drought-Induced Hydraulic Failure: What Traits Matter?

The timing of inception and progression of hydraulic failure can be complicated given it may be driven by impacts associated with plant morphology, biochemistry, and physiology. Nonetheless, the biphasic hydraulic failure framework offers a simplified yet mechanistic approach for locating key traits involved in this process given that this framework is generally supported by empirical evidence (Figure 1B). Below, we list a few key aspects and corresponding traits with a brief discussion about their significance regarding plant drought response as well as current understanding and uncertainties. Of note, one implicit assumption for the development of xylem embolism during this phase is that plants and soil have become hydraulically disconnected prior to the occurrence of embolism in leaves and stems. This assumption is supported by some early studies showing that roots are often more vulnerable to embolism than stems or leaves (Johnson et al., 2016; Wason et al., 2018). However, some recent findings suggest that roots are comparably or even more tolerant to drought-induced embolism compared with other organs in some species (Rodriguez-Dominguez et al., 2018; Peters et al., 2020; Lübbe et al., 2021). Clearly, more studies are required to confirm the spatial pattern of vulnerability to embolism within plants, particularly within the root system and rhizosphere.

### Rooting Depth

Rooting depth delimits the root zone extension of plants in the vertical direction and largely determines plant water acquisition capacity and the availability of water resources to plants. Therefore, rooting depth can significantly affect the response and resilience of plants to drought stress (Canadell et al., 1996), and has long been explicitly considered in most vegetation models, albeit using relatively simplistic mathematical representations (Zeng, 2001). Intuitively, species with deeper rooting systems have a greater chance to capitalize on relatively stable water resources such as deep soil water or groundwater, so there will be less risk of experiencing negative water potential during protracted drought stress. This suggests that the rooting depth trait can be informative in predicting species-specific mortality vulnerability, especially for co-occurring species or individuals showing contrasting drought response (Johnson et al., 2018b; Li et al., 2018b; Jiang et al., 2020). Interestingly, in a study conducted by Wu et al. (2018), the authors noted that the legacy effects of drought, as represented by the offsets between observed growth and predicted growth, persisted longer in deep-rooted trees compared with shallow-rooted functional types such as grasses or shrubs, indicating that deeper roots may compromise drought resilience, therefore questioning the role of deeper roots in facilitating overall fitness. Very few studies have related rooting depth to observed whole plant drought response, mainly due to lack of relevant information regarding this trait. It is well known that plant rooting depth is controlled by many climatic, edaphic, hydrological, and biological factors (Canadell et al., 1996; Fan et al., 2017). However, due to the great difficulty in studying roots, especially roots in the deep soil layer, and the high dynamics of root growth, information regarding rooting depth and its environmental drivers is still very scarce for many species, limiting our ability to better constrain the models. In a synthesis of 2,200 root observations for more than 1,000 species around the globe, Fan et al. (2017) established a hydrologic framework to interpret the spatial variation of plant rooting depth and demonstrated that soil hydrology is a globally common force driving rooting depth patterns from the landscape to global scale. This provides a useful approach to predict plant rooting depth based on the long-term characteristic of soil water profile, which is determined by both precipitation and groundwater level. Yet, the relationship between rooting depth and plant water absorption could become decoupled as the deep soil layer desiccates or the groundwater level declines beyond the capture extent of roots (Xi et al., 2018). Therefore, identifying the environmental and biological drivers for the variation of rooting depth warrants further study.

### Stomatal Regulation

Strictly speaking, stomatal regulation during drought stress is not a trait, although it can be defined by quantitative metrics, which are the dynamics of stomatal conductance (*g*_s_) in response to organ water status (i.e., leaf Ψ; Figure 1, cyan line). However, several key traits can be derived from this response curve and can encompass important implications to model drought-induced tree mortality (Klein, 2014; Blackman et al., 2019; Chen et al., 2019; De Kauwe et al., 2020). Current studies commonly measure g_s_ as a function of leaf Ψ, with the sensitivity of *g*_s_ to drought being quantified by the Ψ at the inception of complete stomatal closure (P_gs_). Theoretically, stomatal closure at less negative water potentials during drought stress would significantly prevent water loss, which in turn reduces the risk of xylem embolism. However, given that stomatal closure also prevents the entry of CO_2_ for carbon assimilation, closing stomata at the expense of carbon gain in the absence of embolism risk will negatively impact the plant and increase the probability of carbon starvation (McDowell et al., 2008). Therefore, water potential at stomatal closure *per se* would be less informative about overall species drought tolerance (Garcia-Forner et al., 2016; Martínez-Vilalta and Garcia-Forner, 2017). In a meta-analysis conducted by Martin-StPaul et al. (2017), the water potential triggering stomatal closure ranges between −1 MPa and approximately −4 MPa across a wide range of species; however, hydraulic safety is largely conferred by more negative water potential thresholds of xylem cavitation, especially for species in arid regions. Therefore, combining stomatal regulation with other traits can often yield metrics that are more informative to plant drought response. For example, by combining water potential thresholds at stomatal closure and xylem cavitation, Skelton et al. (2015) found that the risk of hydraulic failure can be well predicted by the safety margin defined by these two traits. Likewise, in the study of Chen et al. (2019), the percentage of tree survival was correlated with the safety margin based on the P_gs_. Other typical examples include the index of desiccation time proposed by Blackman et al. (2016), which integrates stomatal regulation with many other traits potentially affecting plant drought response and shows great promise in estimating the duration from stomatal closure and mortality. Clearly, quantifiable information on hydraulic safety margin, together with stomatal regulation, is useful to develop better models, yet traits delineating stomatal regulation (e.g., P_gs_) are available for only a few species. Given the difficulties in measuring the response of stomata to drought, extrapolating these values from proper proxies will be instrumental for model parameterization (see below).

### Minimum Conductance

Leaf minimum conductance (*g*_min_) presumably becomes the main route for water leaving the plants, after complete stomatal closure during drought, and hence directly relates to the drought response strategy and survival of the plant (Duursma et al., 2019; Carignato et al., 2020; Lanning et al., 2020). Note that *g*_min_ characterizes the overall water loss rate after stomatal closure under field conditions; therefore, it can be higher than the conductance specifically measured for the adaxial side of leaves (i.e., cuticular conductance, *g*_cut_) and is lower than the conductance value measured from leaves that are dark-adapted or with minimal photosynthetic rate (Duursma et al., 2019). Lower *g*_min_ should confer longer times for dehydration, but studies testing the functional significance of *g*_min_ in the process of drought-related tree mortality are scare. In an experiment designed to test the model described above, Blackman et al. (2019) found no correlation between observed *g*_min_ and observed time to desiccation. However, this does not mean that *g*_min_ plays no role in controlling water loss during dehydration. Instead, water loss during Phase II might be controlled by the conductance of other tissues such as bark. In support, Gleason et al. (2014) showed that desiccation time was significantly correlated with lower rates of transpiration in detached branches, which was the sum of water loss from both leaves and bark. It is also possible that the lack of a relationship was masked by the plasticity of *g*_min_. It has been shown that *g*_min_ is highly responsive to various environmental stimuli, including temperature and water availability (Schuster et al., 2017; Duursma et al., 2019). In Blackman et al. (2019), *g*_min_ may have acclimated to the environment in the common garden, showing only 2-fold variation despite the distinctly different times to mortality across species. More generalizable information regarding the variation in *g*_min_ is required to facilitate model advancement.

### Relative Water Content

Relative water content (RWC) is the classic metric describing the water status of plants. The amount of water in cells governs cell turgor, the maintenance of which is essential for many cell functions. With respect to drought stress, water content in plants directly determines the duration of drought stress that plants can sustain, especially in Phase II when exogenous water supply is no longer available (Blackman et al., 2019). Furthermore, RWC is more than a simple indicator of cell hydration state. Under drought stress, plants need to retain a minimum amount of water to prevent complete cell desiccation. At this stage, the water balance of plants is co-regulated by the capacity of water supply *via* the vascular system, as well as the capacity to retain water in the cell by osmotic regulation; the former depends on xylem embolism resistance, while the latter primarily relies on NSCs (Martinez-Vilalta et al., 2019). The variation is therefore thought to reflect the integration of CS and HF. This concept is highly intriguing as it provides a tool for identifying mortality risk and also allows monitoring tree health at large scales given that RWC can be detected through remote sensing, thus enabling large-scale model evaluation (Konings et al., 2019). However, few studies have tested this concept to date. In Sapes et al. (2019), plant water content scaled linearly with PLC at both the organ and whole plant level in *Pinus ponderosa*, and water content could reflect NSCs content at a given soil water availability. Importantly, a threshold-like function was observed between population mortality risk and plant water content, suggesting the usefulness of this metric in ascertaining the probability of tree death under drought stress.

### Hydraulic Capacitance

Plant hydraulic capacitance (*C*_p_) refers to the water that can be extracted per unit change in water potential. The significance of water storage to whole plant water balance varies among species, and it has been suggested that *C*_p_ could contribute up to 50% of daily water loss from evapotranspiration (Pfautsch et al., 2015). During short-term drought, water stored in the apoplastic, intracellular capillary space or in living cells can be discharged, radially transported to xylem through ray parenchyma, thereby buffering the increasing tension of xylem sap to limit the development of embolism (Richards et al., 2014). In line with this suggestion, a negative correlation between *C*_p_ and the steepness of the xylem vulnerability curve, as represented by the slope of the rapidly increasing PLC phase, has been observed across species (Meinzer et al., 2009). During later phases of drought stress, when *C*_p_ becomes the only water source once water can no longer be taken up from the soil, a larger *C*_p_ should therefore allow plants to live longer. However, experimental evidence supporting this hypothesis is rare and equivocal (Gleason et al., 2014; Blackman et al., 2019). The inconsistent observations among different studies might be attributed to the scale (branch vs. whole plant) or plasticity of the response. For example, there are reports that the *C*_p_ of roots contains a comparable amount of extractable water as stems in some species, and *C*_p_ is also reportedly responsive to environmental moisture (Salomón et al., 2020). In addition, it is also possible that the water provided by *C*_p_ is not utilized to prevent xylem embolism but to sustain the vitality of other tissues such as the cambium (Knipfer et al., 2017, 2019). Nevertheless, *C*_p_ is a measurable trait that can potentially buffer the impact of drought on plant hydraulic status and therefore warrants further studies.

### Leaf Shedding

**Figure 2:**
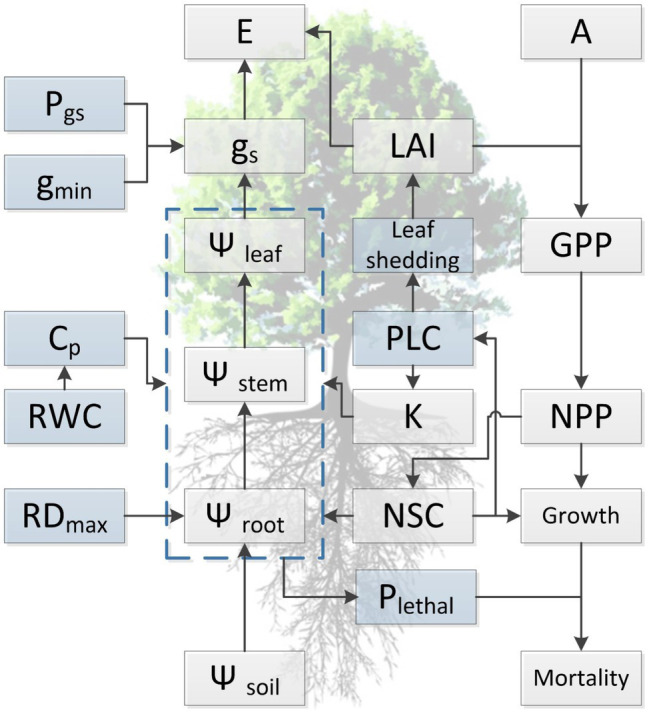
Conceptual diagram summarizing current understanding regarding the relationship among functional traits and illustrating the general approach for simulating of tree dynamics in response to water availability with hydraulic traits in process-based models. Light blue boxes indicate traits that are directly involved in the occurrence of hydraulic failure during drought stress but are not well represented in current TBMs, either due to lack of trait values or insufficient knowledge regarding the variation at spatial or temporal scales. Traits presented in the diagram are maximum rooting depth (RD_max_), water potential of soil, root, stem, and leaf (*Ψ*_soil_, *Ψ*_root_, *Ψ*_stem_, and *Ψ*_leaf_, respectively), relative water content (RWC), hydraulic capacitance (*C*_p_), minimum conductance (*g*_min_), water potential threshold of stomata respond to drought (P_gs_), hydraulic conductivity (K), percentage loss of hydraulic conductivity (PLC), stomatal conductance (*g*_s_), evaporation (E), leaf area index (LAI), leaf photosynthesis (A), non-structural carbohydrates (NSCs), lethal threshold (P_lethal_), gross (GPP), and net primary productivity (NPP).

Plant water loss primarily occurs through leaves; therefore, adjustments in canopy leaf area have significant influence on water balance of plants. Drought deciduous plants undergo annual cycles of leaf shedding and flushing in response to regular seasonal drought; this phenological strategy allows deciduous trees to avoid water stress associated with the dry season. However, even in evergreen species prolonged drought stress can trigger leaf shedding (Figure 1, dashed yellow line). This phenomenon, commonly termed as hydraulic segmentation, is hypothesized to reduce the risk of xylem embolism given that water loss can be significantly minimized once leaves have been hydraulically isolated from the plant. Studies of hydraulic segmentation commonly focus on revealing its underlying mechanisms by identifying differences in vulnerability to embolism or hydraulic resistance across organs (Pivovaroff et al., 2014; Levionnois et al., 2020). It has been shown that embolism resistance or hydraulic resistance varies across organs in some species, but not in others, indicating that leaf shedding is not a universal strategy (Zhu et al., 2018). On the other hand, the effects of leaf shedding on plant water status are less well understood. Incorporating a leaf shedding function into models significantly increased the time to mortality and reduced the offset between predicted and observed values, suggesting that leaf area adjustment does facilitate water retention during drought stress (Blackman et al., 2019). However, the functional significance of this strategy may be debatable for some species exhibiting leaf shedding during drought stress. For instance, Wolfe et al. (2016) reported that the occurrence of leaf shedding coincided with a 50% loss of maximum xylem conductivity. Of note, one of the studied species, *Genipa americana*, showed continuously decreasing water potential after leaf shedding, indicating that leaf area adjustments may not always stabilize plant water status, if water loss with continuous drying is dominated by other organs. Similarly, in the study of Blackman et al. (2019), leaf shedding was initiated at water potentials triggering 50% loss of hydraulic conductivity in stems (P_50_), suggesting that reducing canopy leaf area may have limited capacity to prevent the occurrence of xylem embolism (Figure 2).

### Lethal Threshold

Given that hydraulic failure occurs due to massive xylem cavitation, it is intuitive that there is a lethal threshold in xylem vulnerability that generates a fatal degree of embolism. By subjecting plants to drought and then a re-watering treatment, Brodribb and Cochard (2009) showed that once the xylem of several gymnosperms lost 50% of the maximum hydraulic conductivity, recovery did not occur upon re-watering. Based on this finding, they suggested that the P_50_ of stems can be used as the lethal water potential threshold (P_lethal_) for this functional group. Using the same approach, Urli et al. (2013) found that the P_lethal_ in angiosperms corresponds to the water potential threshold triggering 88–100% loss of xylem hydraulic conductivity (P_88_). These thresholds are generally valid for whole plants and organs such as leaves (Blackman et al., 2009; Figure 1, P_lethal_). However, uncertainty remains regarding the commonality of these water potential thresholds. Common approaches for determining P_lethal_ are often time-consuming; therefore, these threshold water potentials are derived from limited species. By compiling existing P_lethal_ data, Liang et al. (2021) recently showed that stem P_50_ and P_88_ explained 75 and 43% of the variation in P_lethal_ across gymnosperm and angiosperm species, respectively. In particular, lethal water potentials of some angiosperm species are much lower than P_88_, as have been observed by Li et al. (2016). Tree death occurs under drought because living cells are dehydrated and therefore, trees are unable to regain metabolic function by reconstructing damaged tissues even after drought stress has been alleviated. Given the observed discrepancy between embolism threshold and P_lethal_, it is likely that drought tolerance of some species can be organization level-specific, such that cell vitality can persist longer than the integrity of vascular water transport under drought stress. Therefore, assigning P_lethal_ based on cellular hydraulic failure represents a more mechanistic and robust approach, yet the vulnerability of cell vitality to dehydration remains unclear.

## Toward a Better Representation of Tree Drought Mortality in Models With a Mechanistic Approach

Providing a mechanistic underpinning to better simulate drought mortality in trees is not an easy endeavor, as described above, but data-driven evidence is emerging and has led to an increasing momentum toward incorporating plant hydraulic traits in LSMs. Previously, predictive models often relied on empirical relationships to introduce soil moisture stress on plants and to induce the subsequent tree death (De Kauwe et al., 2015; Medlyn et al., 2015, 2016; Christoffersen et al., 2016; Trugman et al., 2018). Clearly this approach is not very applicable for novel conditions, which often change rapidly over space and time with global climate change. The abovementioned hydraulic traits and the associated ecophysiological processes offer a framework through which a more mechanistic simulation of drought mortality can be realized.

Indeed, many LSMs have incorporated some of these hydraulic traits and processes into their modeling framework. These models are often conceptually similar, i.e., plant hydraulics exert control on growth through leaf gas exchange, and tree mortality occurs once P_lethal_ is approached (Figure 2) but can differ in model structure and parameter. McDowell et al. (2013) evaluated six models of different scales using a data-model inter-comparison and highlighted that the capacity to simulate internal hydraulic and carbohydrate dynamics is crucial to capture observed tree mortality in the models. More recent modeling exercises showed a similar role for plant hydraulics. For example, De Kauwe et al. (2015) replaced the empirical scalar parameter (i.e., beta) for drought stress with a new expression for drought sensitivity of gas exchange that depends on leaf water potentials (i.e., Zhou et al., 2013, 2014) in the CABLE model. This new model feature led to improved simulation of gross primary production during the drought period, especially at the more xeric sites along a rainfall gradient (De Kauwe et al., 2015). Similarly, Xu et al. (2016) introduced a trait-driven plant hydraulic module into the ED2 model, which led to more realistic predictions of plant hydraulic dynamics, such as leaf water potential and stem sap-flow and better spatial predictions of leaf area index. Kennedy et al. (2019) implemented a plant hydraulic stress configuration to the Community Land Model (CLM5) that better connects stomatal conductance to water stress *via* the influence of vegetation water potential, allowing hydraulic redistribution and compensatory root water uptake to buffer shortfalls in rainfall (Kennedy et al., 2019). All these abovementioned model developments regarding plant hydraulics could have significant implications for the simulated land-climate feedback, thereby providing crucial benefits to better anticipate cascading climate change consequences. Nonetheless, few LSMs have explicitly integrated current state-of-art knowledge regarding the development of hydraulic dysfunction into modeling due to existing data uncertainties for many traits mentioned above. We suggest that advancing our mechanistic understanding and building sufficient datasets, that allow traits variation at spatial or temporal scales to be predicted, will help to reduce these uncertainties (Table 1). Overall, this will facilitate data-model integration and improve the representation of drought-induced tree mortality in LSMs.

**Table 1:** Key summary of plant hydraulic traits and/or variables and their model integration recommendations.

Measurable trait/variable	Definition	Drought-related functionality	Data-model integration recommendations	Data uncertainty to support model integration
RWC	Relative water content in plant tissue	Affects plant hydraulic capacitance and plant drought tolerance	Need to generalize the functional relationship to guide model development, possibly *via* a variable RWC (within bound) in response to environmental perturbation	High
RD_max_	The maximum rooting depth	Affects plant water uptake from the soil and plant drought tolerance	Refine PFT-specific parameter in the model to better reflect plant and landscape heterogeneity	Low
ψ	Water potential of major plant tissue	Affects plant hydraulic conductivity and water loss *via* stomatal conductance, also affects plant carbon uptake and transport	Incorporate the functional relationship into models, in particular those plant hydraulic vulnerability and carbon status, and collect model evaluation datasets	Moderate
*C_p_*	Plant hydraulic capacitance: the amount of water that can be extracted per unit change in ψ	Affects plant xylem vulnerability and plant hydraulic conductivity	Incorporate this parameter to better reflect its functional effect in the model	Moderate
*K*	Whole plant hydraulic conductance	Determines the capacity for plant to transport water during drought	Develop datasets to generalize its functional effect	High
*g*_min_	Leaf minimum stomatal conductance	Characterizes plant water loss *via* leaf after stomatal closure during drought	Incorporate this parameter in the model and allow decoupling between carbon assimilation and stomatal conductance during drought	Moderate
P_gs_	Leaf water potential at the inception of complete stomatal closure	Affects plant hydraulic conductivity, stomatal water loss and plant desiccation time during drought	Collect this parameter together with *g*_min_ and integrate into the functional relationship of plant hydraulics in the model	Moderate
P_50_	50% loss of xylem conductivity	Affects plant hydraulic conductivity and desiccation time	Develop a dataset and integrate into the functional relationship of plant hydraulics in the model	Moderate
P_88_	88% loss of xylem conductivity	Affects plant hydraulic conductivity and desiccation time	Develop a dataset and integrate into the functional relationship of plant hydraulics in the model	Moderate
P_lethel_	Lethal threshold of loss of xylem conductivity	Indicates plant mortality	Develop a dataset and integrate into the functional relationship of plant hydraulics in the model	Moderate

*Note that the data uncertainty to support model integration is a qualitative assessment based on available literature and thus should be considered in caution. PFT denotes plant-functional group*.

## Expanding the Trait Database Using Easily Measured Proxies

A critical pre-requisite of process-based modeling is the information regarding traits values and the pattern of variation. Global databases of functional traits are becoming increasingly available but are often constrained to limited trait types and species (Kattge et al., 2020). This poses a major obstacle to its application, especially when predicting drought-induced tree mortality, the occurrence of which is likely ubiquitous across diverse tree species and is often a function of various traits (Blackman et al., 2016; Choat et al., 2018; Blackman et al., 2019; Chen et al., 2021a). With regard to plant hydraulics, many of the fundamental traits engaged in the process of hydraulic failure are only available for a few hundred species (Bartlett et al., 2012; Choat et al., 2012; Klein, 2014; Bartlett et al., 2016), which accounts for a trivial portion of the vast plant taxa. One important reason for the lack of sufficient data is that traits representing plant hydraulics are usually difficult to measure. Assessments of key functional traits (e.g., thresholds for stomatal closure, xylem embolism, or tree death) are often time- and labor-consuming, and can often be hard to realize under field conditions. Although new techniques are emerging to address these issues (Gauthey et al., 2020; Chen et al., 2021b), building substantial datasets still requires considerable time.

In plants, many functional traits are mathematically correlated across species. Positive correlation can arise because of shared ancestry, functional convergence, or subjected to co-selection, while negative correlative relationships can result from functional or structural trade-offs (Wright et al., 2007; Reich, 2014; Bartlett et al., 2016). Such correlative relationships not only provide insights into many key ecological questions, such as principles governing community assembly or species coexistence, but also offer the opportunity to rapidly gain information regarding traits variation across diverse species. Using easily measured traits (i.e., soft traits), as proxies of traits that are difficult to measure (i.e., hard traits), has become a common approach in traits-based ecology, especially when TBMs are parameterized (Rogers et al., 2017). Importantly, these relationships often reflect mechanistic implications rather than demonstrated causality (Reich et al., 1998).

**Figure 3:**
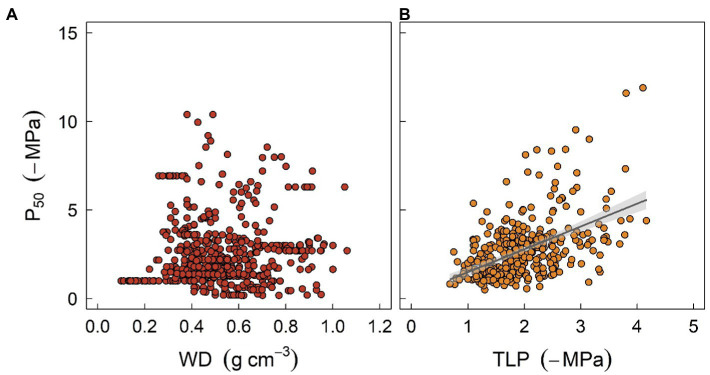
The relationships between stem water potential at 50% loss of xylem hydraulic conductivity (P_50_) and two easily measured traits, sapwood density (WD, panel **A**), and leaf turgor loss point (TLP, panel **B**), at global scale. P_50_ data were obtained from Choat et al. (2012). Data for WD were sourced from Zanne et al. (2009), and TLP data were compiled from Bartlett et al. (2012) and Zhu et al. (2018). Regression formula for panel **(B)** *y* = 1.34*x* + 0.25 (*R*^2^ = 0.22, *p* < 0.001).

With respect to plant water relations, coordination and trade-offs have been found among various hydraulic or structural traits. Among others, woody density (WD), which is thought to integrate a suite of plant functions, has been shown to be a strong predictor of xylem vulnerability to embolism (Markesteijn et al., 2011; Li et al., 2018a; Liang et al., 2021). Furthermore, wood density is correlated with sapwood hydraulic capacitance, which may facilitate both drought resistance and resilience (Li et al., 2018a; Santiago et al., 2018). It has been observed that species with high wood density are more resistant to drought-induced xylem embolism. Mechanisms underpinning this observation may be due to reinforced vessel walls, which can resist xylem implosion and therefore cavitation (Hacke et al., 2001), although collapse of xylem vessels is seldomly reported in stems. The correlation between WD and xylem embolism resistance has been adopted by some models. For instance, in the work of Xu et al. (2016), WD was used to predict the water potential threshold of xylem embolism, and the latter was then used to parameterize the hydraulic module of ED2. However, caution should be taken when extrapolating this correlation to assess mortality risk for field-grown trees given that the two traits are not always related (Figure 3A). For example, some species characterized by high WD tend to exhibit a higher mortality ratio during drought stress (Hoffmann et al., 2011). One possible reason is that WD is not consistently correlated with embolism resistance. Indeed, high WD can be supported by features that are unrelated to plant hydraulics, such as fiber traits (Russo et al., 2010). Additionally, the occurrence of hydraulic failure, as discussed above, is a function of multiple traits. A species characterized by embolism-resistant xylem can still die rapidly during drought stress due to a profligate water regulation strategy or water acquisition ability (Hoffmann et al., 2011; Fu et al., 2019).

Another valuable trait is leaf water potential at turgor loss point (TLP), which has been used to assess drought tolerance in plant physiology for decades (Bartlett et al., 2012). TLP is often correlated with water potential thresholds triggering stomatal closure and xylem embolism (Figure 3B), either because of mechanistic linkages given that stomatal closure is structurally driven by the turgor loss of guard cells, or functional linkages as less resistance xylem typically requires early stomatal closure to minimize water loss (Buckley, 2019). Recent findings also suggest that TLP integrates a wide range of hydraulic traits and thereby describes the overall water use strategy of plants (Meinzer et al., 2016; Fu et al., 2019; Li et al., 2019). Furthermore, it has been shown that TLP can also be correlated with economic traits such as specific leaf area, photosynthetic rate, or leaf longevity, although the correlations can be much weaker (Zhu et al., 2018), suggesting that diverse aspects of plant strategy can probably converge to a simple trait (Reich, 2014). Indeed, several meta-analysis studies have shown that TLP varies systematically with site water availability within and across biomes, indicating that TLP does provide some adaptive advantages (Bartlett et al., 2012; Zhu et al., 2018). However, the correlation between TLP and traits such as xylem embolism threshold is still surprising as TLP is known to be highly plastic (Farrell et al., 2017; Johnson et al., 2018a), while embolism threshold is more conservative within and across species (Lamy et al., 2014; Li et al., 2018a). Again, one should be cautious when using TLP as a surrogate given that it can easily become decoupled from other hydraulic traits due to growth conditions. Overall, soft traits like WD or TLP provide a rapid tool for generating other traits essential for model parameterization, as well as assessing plant strategy for a considerable number of species. Nonetheless, “true” values of hard traits are warranted to increase the precision of model outcomes.

## Pathway Forward

The elusive nature of physiological mechanisms underpinning drought-induced tree mortality suggest that detailed biological processes and their interactions from the beginning of drought response to complete desiccation can be difficult to model. Nonetheless, the identification of hydraulic dysfunction as a major driver of tree death during drought stress, together with the biphasic framework depicting the development of xylem embolism, allows us to locate key functional traits involved in this complicated phenomenon and to predict its occurrence using a mechanistic approach. To better represent hydraulic failure in process-based models, we suggest that the following areas require future attention.

Firstly, physiological mechanisms for plant water regulation strategy during drought stress need to be better elucidated. Drought-induced tree death occurs when plant water potential approaches a critical threshold where xylem water transport is largely blocked by embolism. To prevent water potential from reaching this tipping point, plants would adopt diverse regulation strategies to maintain a reasonable water status. However, many of these strategies are not well represented in current models. For example, hydraulic segmentation has long been thought to mitigate the decline of water potential. Yet, the physiological basis of this phenomenon remains unclear. Hypotheses explaining leaf shedding during drought stress include differences in vulnerability to embolism or hydraulic resistance across organs, both of which are supported by experimental evidence, but can generate distinct ecological consequences. A similar example is xylem refilling, which still represents a research frontier in the field of plant hydraulics. Whether an embolized vessel can hydraulically recover after drought is still controversial. Several mechanisms have been proposed, but it remains unknown whether it is common in species, the nature of the circumstances leading to xylem refilling, and potential impacts on water transport. Identification of these physiological mechanisms is crucial because it determines how this strategy will be represented and parameterized in models.

Secondly, functional traits influencing water transport of plants need to be fully examined, especially for traits governing belowground hydraulic processes. Although data of key hydraulic traits such as vulnerability to embolism are rapidly accumulating, information regarding other functional traits affecting plant hydraulics is limited. With respect to aboveground water relations, traits, such as cuticular conductance, hydraulic capacitance, or water potential threshold for stomatal closure, are only available for a relatively small number of species compared with embolism thresholds. In addition, trait variation with the environment is also largely unknown. Likewise, belowground traits affecting water uptake and conductance are limited. Root functional traits that can be measured with relatively simple approaches should be the focus, such as maximum rooting depth, vertical root distribution, proportions of fine (absorption) and coarse (transport) roots, and water uptake vulnerability to soil desiccation. In addition, the variability of root traits with tree age should be determined to facilitate assessment of impacts in a dynamic way.

Patterns and sources of intraspecific trait variation at spatial and temporal scales require further investigation. The inter-specific variation of traits related to water regulation has been extensively studied and many of these traits vary systematically along an aridity gradient. However, the pattern of intraspecific trait variation is less clear, with the environmental drivers somewhat inconclusive. In some species, the variation of hydraulic traits among populations shares a similar pattern as variation across species, i.e., traits generally shift toward an increased drought tolerance as habitat water availability becomes limited. However, in other species, hydraulic traits can be highly conservative despite variation in environmental dryness. In addition, traits may also vary over time for a given population, but studies focusing on this aspect are infrequent. It has been shown that some hydraulic traits, including vulnerability to embolism or hydraulic capacitance, can shift seasonally or interannually (Guo et al., 2020; Sorek et al., 2021; Wu et al., 2021), leading to temporally altered overall water regulation strategy. This type of variation can be due to differences in ontogeny or shifts in environmental conditions over time. A useful framework for understanding observed differences in traits within species is to separate the observed difference into genotype variation and phenotypic plasticity and to unravel the source of variation using common garden experiments. If trait expression is governed by genetics, then we would expect small differences in targeted traits across populations, and we could apply a single trait value in models, regardless of the population-specific growth conditions. However, if phenotypic plasticity is dominant, then controlled environment experiments or transect studies in the field will be needed to uncover the environmental driver and then calculate the trait value as a function of environmental variables in models.

## Conclusion

In conclusion, drought-related tree mortality is a ramification of complex interactions, consisting of multiple physiological processes associated with carbon and hydraulic dynamics. Recent advancements in plant physiology indicate the crucial role of hydraulic failure in determining tree mortality under drought, which offers a pathway for LSMs to represent mortality events with a simplified, but efficacious approach. In particular, the biphasic framework describing the development of hydraulic failure is generally supported by current experimental evidence and is therefore instrumental in locating key traits. The incorporation of these key traits will have the potential to boost the predictability of tree mortality under drought, without introducing much complexity to the algorithms and burdening the computational capacity. Nonetheless, many hydraulic processes within this framework are not mechanistically understood or remain controversial, which represents the priority of future research in the field of plant hydraulics. In addition, traits coordination and variation at different levels (e.g., intra- and inter-specific) and scales (i.e., spatial and temporal) also warrant further study, which would facilitate model parameterization by providing and constraining trait values.

## Author Contributions

XL drafted the early version of this manuscript, with inputs from BX on root hydraulics and MJ on ecosystem models. DT provided critical edits. All authors contributed to the article and approved the submitted version.

## Funding

XL is financially supported by research initializing funding from Minzu University of China (nos. 0210040217 and 2020CXTD). BX is supported by the National Natural Science Foundation of China (no. 32171763). JF receives funds from the interdisciplinary research program of Minzu University of China (nos. 2020MDJC09 and 2021XSTD02). MJ acknowledges funding from the Australia Research Council (DE210101654).

## Conflict of Interest

The authors declare that the research was conducted in the absence of any commercial or financial relationships that could be construed as a potential conflict of interest.

## Publisher’s Note

All claims expressed in this article are solely those of the authors and do not necessarily represent those of their affiliated organizations, or those of the publisher, the editors and the reviewers. Any product that may be evaluated in this article, or claim that may be made by its manufacturer, is not guaranteed or endorsed by the publisher.
