# Corrigendum: Unlocking drought-induced tree mortality: Physiological mechanisms to modelling

**DOI:** 10.3389/fpls.2022.1126049

**Published:** 2023-01-09

**Authors:** Ximeng Li, Benye Xi, Xiuchen Wu, Brendan Choat, Jinchao Feng, Mingkai Jiang, David Tissue

**Affiliations:** ^1^ College of Life and Environmental Science, Minzu University of China, Beijing, China; ^2^ Hawkesbury Institute for the Environment, Western Sydney University, Richmond, NSW, Australia; ^3^ Ministry of Education Key Laboratory of Silviculture and Conservation, Beijing Forestry University, Beijing, China; ^4^ State Key Laboratory of Earth Surface Processes and Resource Ecology, Beijing Normal University, Beijing, China; ^5^ College of Life Sciences, Zhejiang University, Hangzhou, China; ^6^ Global Centre for Land-based Innovation, Western Sydney University, Richmond, NSW, Australia

**Keywords:** drought, tree mortality, hydraulic failure, carbohydrates, functional traits, plant hydraulics, land surface models

In the published article Duursma et al. (2019) was not cited. The citation has now been inserted in ‘Parameterizing Drought-Induced Hydraulic Failure: What Traits Matter?, Minimum Conductance’:

“It has been shown that g_min_ is highly responsive to various environmental stimuli, including temperature and water availability (Schuster et al., 2017; Duursma et al., 2019)”

The authors apologize for this error and state that this does not change the scientific conclusions of the article in any way. The original article has been updated.

